# Reply to “High Quantitative Accuracy Is Possible with ^177^Lu SPECT”

**DOI:** 10.2967/jnumed.126.272772

**Published:** 2026-10

**Authors:** Bella Salerno, Mercy Akerele, Daniel L.J. Thorek

**REPLY:** We thank Dewaraja, Dickson, and Tran-Gia for their thoughtful engagement with our work and welcome the opportunity for this exchange. The question of quantitative rigor in nuclear medicine imaging is one of the most consequential methodologic debates in the field. As theranostic programs using ^177^Lu, ^90^Y, and ^223^Ra, along with emerging agents such as ^225^Ac, ^161^Tb, and ^203^/^212^Pb, expand rapidly into the clinical sphere, the standards to which dosimetry-relevant imaging must be held become a matter of patient safety and efficacy. Dose–response relationships that underpin treatment personalization are meaningful only if the quantitative foundation on which they rest has been rigorously characterized. It is therefore precisely this kind of critical scrutiny that the field requires.

The letter highlights the ^177^Lu data and analysis, the difference between these and ^203^Pb data, and methodology reporting in our article (1). The goals of our study were to perform a controlled, cross-modality comparison under consistent acquisition conditions, rather than to investigate the best case or optimized performance for each system. Indeed, we demonstrated the impact of several SPECT reconstruction factors on the acquired data and chose parameters that aligned with our routine clinical imaging practices. As we noted, we were inspired by the efforts of Ryu et al. (2) to apply NEMA NU 2 methodology to SPECT acquisitions. This framework has been established to objectively compare the image quality, sensitivity, and accuracy of different PET imaging systems, and is lacking for single-photon imaging approaches. On the issue of ^177^Lu imaging, we note that the values in Ryu et al.’s study and ours align in the context of ^177^Lu activity recovery. We agree that these recovery coefficients (RCs) fall on the lower end of the reported range; however, they reflect a baseline performance before any optimized vender-specific workflows are applied. Importantly, our findings, as well as those in the broader literature (2–4), show that there is a wide range of reported values, demonstrating the need for standardized approaches, which was another substantial point of our report.

We also want to emphasize that RCs are not an absolute measurement of quantitative reliability for a system. Depending on volume-of-interest segmentation, corrections, reconstructions, counts, and count conversion methods, along with many more factors, one may produce differentiated results. Therefore, RCs, especially for SPECT, are more representative of methodologic implementation and not necessarily intrinsic system capabilities. That being said, this underlies our rationale for the study in the fact that there is no “gold standard” for quantitative SPECT evaluation. This raises the broader challenge of ensuring that count values derived from imaging accurately reflect true activity concentrations, given the multiple corrections applied and the substantial photon loss before detection.

A clear example of the sensitivity of SPECT quantification to minor variations in imaging protocols is demonstrated by the authors’ own recent study in which they have shown that object positioning can lead to measurable differences in RCs (5). Indeed, we have found similar effects at our center, which reinforce the challenges associated with the use of SPECT as a quantitative imaging tool without rigorous standardization and analytic considerations. We note that this aberration is not observed in PET (Supplemental Fig. 1, available at http://jnm.snmjournals.org). This further supports a conclusion of our work that, when quantitative accuracy is paramount, PET should be preferred because it offers more consistent performance.

We also would like to address the comments related to the discrepancies between ^203^Pb and ^177^Lu results. In our analysis, spatial resolution was evaluated as a function of sphere diameter, and all SPECT isotopes demonstrated deviations from the ideal relationship between measured and true sphere size, indicating inherent resolution limitations. Notably, ^177^Lu showed the greatest deviation from the ideal trend at the largest sphere diameter (37 mm). Additionally, for the smallest sphere, ^177^Lu and ^203^Pb had similar measurements. It should not be assumed that ^177^Lu would necessarily exhibit better RC performance than ^203^Pb. Although ^177^Lu has lower γ-energy, its photon abundance is also substantially lower (208 keV at 10.4%) compared with ^203^Pb (279 keV at 80.9%). Because sufficient counting statistics are critical for accurately recovering true activity values, the lower photon yield of ^177^Lu can limit quantitation and contribute to the poorer RC performance.

Our conclusion that SPECT demonstrates lower quantitative accuracy compared with PET under the same conditions is intended to be context-dependent, not absolute. SPECT demonstrates lower quantitative accuracy compared with PET under the same conditions. SPECT remains highly valuable clinically, but continued efforts toward harmonized and consistent analysis pipelines are needed, particularly given the wide variability reported even in the most geometrically simplistic, standardized phantom studies.

We share the correspondents’ aspiration for a future in which ^177^Lu SPECT delivers consistent quantitative accuracy across clinical sites and systems. Our study reflects performance under conditions that are representative of a range of clinical deployments. The harmonization and standardization efforts they cite represent genuine and important progress. However, this progress remains incomplete. State-of-the-art performance requires careful calibration, resolution modeling, optimized scatter correction, and site-specific phantom validation. We are excited to see that these important points are being considered in research laboratories, in the clinic, and by manufacturers in order to bring us closer to realizing this aspiration.

## DISCLOSURE

No potential conflict of interest relevant to this article was reported.

## Notes

**Bella Salerno**

*School of Medicine, Washington University in St. Louis, St. Louis, Missouri*

**Mercy Akerele**

*School of Medicine, Washington University in St. Louis, St. Louis, Missouri*

**Daniel L.J. Thorek**

*School of Medicine, Washington University in St. Louis, St. Louis, Missouri*

*E-mail: thorekd@wustl.edu*
